# Mouse Oocytes, A Complex Single Cell Transcriptome

**DOI:** 10.3389/fcell.2022.827937

**Published:** 2022-03-07

**Authors:** Di Wu

**Affiliations:** Laboratory of Cellular and Developmental Biology, NIDDK, National Institutes of Health, Bethesda, MD, United States

**Keywords:** RNA-seq, normalization, RNA degradation, single oocyte RNA-seq, germinal vesicle oocytes, mouse oocyte maturation

## Abstract

Germinal vesicle (GV) stage is a critical transition point from growth to maturation in mammalian oocyte development. During the following meiotic maturation, active RNA degradation and absence of transcription significantly reprofile the oocyte transcriptome to determine oocyte quality. Oocyte RNA-seq has revealed transcriptome differences between two defined phases of GV stage, namely non-surrounded nucleolus (NSN) and surrounded nucleolus (SN) phases. In addition, oocyte RNA-seq has identified a variety of dysregulated genes upon genetic mutation or environmental perturbation. Historically, due to the low amount of RNA per oocyte, a few (20–200) oocytes were needed for a regular library construction in bulk RNA-seq. In recent years, development of single cell sequencing allows detailing the transcriptome of individual oocytes. Here in this study, different RNA-seq datasets from single and bulk of mouse oocytes are compared, and single oocyte RNA-seq (soRNA-seq) shows higher reproducibility. In addition, soRNA-seq better illustrates developmental progression of GV oocytes, revealing more complex gene changes than traditional views. Specially, an elevated level of ribosomal RNA 5′-ETS (5′ external transcribed spacer) has been shown to highly correlate with SN property. This study further demonstrates that UMI (unique molecular identifiers) based and other deduplication methods are limited in their ability to improve the precision of the soRNA-seq datasets. Finally, this study proposes that external spike-in molecules are useful for normalizing samples of different transcriptome sizes. A list of stable genes has been identified during oocyte maturation that are comparable to external spike-in molecules. These findings highlight the advantage of soRNA-seq, and have established ways for better clustering and cross-stage normalization, which can provide more insight into the biological features of oocyte maturation.

## Introduction

Oocyte meiotic maturation immediately determines oocyte quality, during which around 70% of oocyte RNA undergoes degradation (Piko and Clegg, 1982; Yu et al., 2016a; Wu and Dean, 2020). Ablation of RNA degradation results in oocyte arrest, infertility, and abnormal early embryogenesis (Yu et al., 2016a; Dumdie et al., 2018; Wu and Dean, 2020). Germinal vesicle (GV) stage initiates meiotic maturation by terminating transcription, preparing for chromatin compaction and nuclear envelope breakdown. Nuclear staining has identified two subpopulations of GV oocytes, namely non-surrounded nucleolus (NSN) and surrounded nucleolus (SN) based on their nuclear configurations. Bulk RNA-seq and integrated genetic studies suggest SN oocytes are more competent for embryogenesis (Ma et al., 2013; Schultz et al., 2018).

Single cell RNA-seq has revolutionized investigations of transcriptomes that transform cell fate during development. As a single cell, the mammalian fully-grown oocyte is unique in its large size, uniform morphology, high nucleic acid content and numerous types of RNA. Since single oocyte RNA-seq (soRNA-seq) was introduced, it has become increasingly popular for multi-omic studies in mice (Tang et al., 2010). These advances provide greater understanding of developmental and genetic variation. In addition, multi-omic studies also detailed information in the co-regulation of different types of transcripts that affect oocyte quality and ageing, such as protein-coding RNA, small RNA and ribosomal RNA, etc (Dumdie et al., 2018; Schultz et al., 2018; Chen et al., 2019; Mihalas et al., 2019; Wu and Dean, 2020). However, there has been no direct evaluation of soRNA-seq compared to bulk oocyte RNA-seq, or any discussion of different analytical methods.

The current study firstly aimed to do a thorough comparison among single and bulk oocyte RNA-seq datasets. By pairwise analyses of several published RNA-seq datasets, soRNA-seq exhibited higher reproducibility. Secondly, the variation of soRNA-seq data were shown to reveal more complex transcriptome differences of morphologically similar GV oocytes (e.g., SN, NSN and intermediate) than previous views. Interestingly, a strong correlation of 5′-ETS elevation with potential SN phase has been discovered. Thirdly, by performing multiple deduplication strategies, there was high similarity in downstream analyses either with or without deduplication. Finally, it was demonstrated that external spike-in molecules, such as ERCC (External RNA Control Consortium), can effectively account for transcriptome size changes during oocyte maturation, during which active RNA degradation takes place without transcription. For situations when ERCC is not available, a group of stably transcribed genes (constGenes) during oocyte maturation was extracted, which provided high similarity to ERCC for cross-stage normalization. This normalization allows greater appreciation of oocyte heterogeneity at GV stage and across maturation. These observations shed light on future oocyte transcriptomic studies.

## Materials and Methods

### Oocyte Collection and Culture

Ovaries were dissected in PBS, and transferred into M2 medium (CytoSpring, M2114) plus milrinone (2.5 μM). The ovaries were pierced mechanically with a 30-gauge needle to release oocytes and only fully-grown oocytes (nuclear envelope-intact oocytes) were collected for further experiments.

### RNA-Seq Library Preparation of Intact and Fractions of Mouse Oocytes

Individual and fractions (1/2, 1/4, 1/8) of mouse GV oocyte RNA-seq libraries were prepared according to a published pipeline with minor modifications (Macaulay et al., 2016; Wu and Dean, 2020). Briefly, single GV oocytes from a healthy B6D2_F1_ female at 12 weeks old were collected and transferred individually into 2.5 μl RLT Plus (Qiagen) and stored at −80°C. A single GV oocyte lysis was diluted 1:2, 1:4 and 1:8 which represented fractions of oocytes. In total, eight oocytes were used for all libraries, including six for different amplification cycles and two for fractional oocytes. To prepare libraries for sequencing, 1 μl of the 10^5^-fold diluted ERCC spike-in mix (Thermo Fisher Scientific, 4456740) was added to 2.5 μl of each single or fractional oocyte sample. Poly(A) RNA was isolated by oligo (dT) beads, reverse transcribed, amplified and purified (Macaulay et al., 2016). Different fractions of oocytes (1/8, 1/4, 1/2, 1) were amplified for 18 cycles. After purification, cDNAs were evaluated by Bioanalyzer 2,100 (Agilent). Qualified cDNAs were used to construct sequencing libraries by Nextera DNA Sample Preparation Kits (Illumina). The sequencing was performed by the NIDDK Genomic Core Facility using the HiSeq 2,500 Sequencing System (Illumina).

### Use of Published RNA-Seq Datasets

15 published mouse oocyte RNA-seq datasets, including 121 libraries (containing bulk and single) are used in the current study (Supplementary Table S11). Only wildtype (control group) oocytes from the listed datasets are used here, because the mutant oocytes and aged oocytes exhibited significant differences in their transcriptomes compared to control groups, which made them improper for comparing reproducibility. All datasets are additionally named by whether using single (s) or bulk (b) mouse oocytes as the initial material, and the library construction methods (pA or rM). Specifically, the library construction protocols (or kits) were checked for each dataset: all libraries that used oligo (dT)-mediated RNA isolation and/or reverse transcription are considered as poly(A)-based (pA); all libraries that used ribosomal RNA probe-mediated rRNA clearance are considered as RiboMinus (rM). The single oocyte RNA-seq datasets do not contain original SN or NSN information. The SN and NSN feature genes were obtained from a bulk RNA-seq result (Ma et al., 2013) and implemented in analysis as described below.

### RNA-Seq Analysis

SRA files of all datasets (Supplementary Table S11) were downloaded from NCBI and converted to Fastq files using fasterq-dump tool of the SRA Toolkit (v2.11.2). Reads from each Fastq file were trimmed with Cutadapt (v3.4) for light quality trimming with parameters “-m 10 -q 20, 20”. The number of reads that are aligned to coding, UTR, intronic or intergenic regions were calculated using Picard tools CollectRnaSeqMetrics (v2.25.7). The trimmed reads were mapped to the *Mus musculus* GRCm38 genome plus ERCC. fasta using STAR (v2.7.8a) to get the Bam files.

The Bam files were processed differently for downstream analyses in Original, Dedup and Picard groups. For the Original group, reads were counted using HTSeq (v0.11.4) with default parameters. For the Dedup reads group, before reads counting, the Bam files were processed for deduplication using the UMI index reads and alignments according to the Ovation Solo RNA-seq manual v4 (python nudup. py). For the Picard reads group, the Bam files were pre-processed by Picard tools (v2.25.7) to mark and remove the duplicated reads without implementing the UMI index reads. The deduplicated Bam files were then counted using HTSeq (v0.11.4) with default parameters. Count files were used for differential analysis.

Differential expression between groups was analyzed using R (v3.5.1) with DESeq2 (v1.24.0) using default parameters. A gene/ERCC was considered valid to be included in differential analysis when it had at least five reads in at least two libraries. In all cases, significantly changed genes are defined as *P-adjust* < 0.01. Principal component analysis and k-means clustering were performed using the factoextra R package (v1.0.3). In all k-means clustering, the value of nstart was set as 25 to get the best one among 25 initial configurations.

When ERCC molecules or constGenes were used for normalization, the counts of the ERCC molecules or constGenes were provided as the “controlGenes” for estimating the size factors. Otherwise, the default estimation method (median ratio) was used.

**FIGURE 1:**
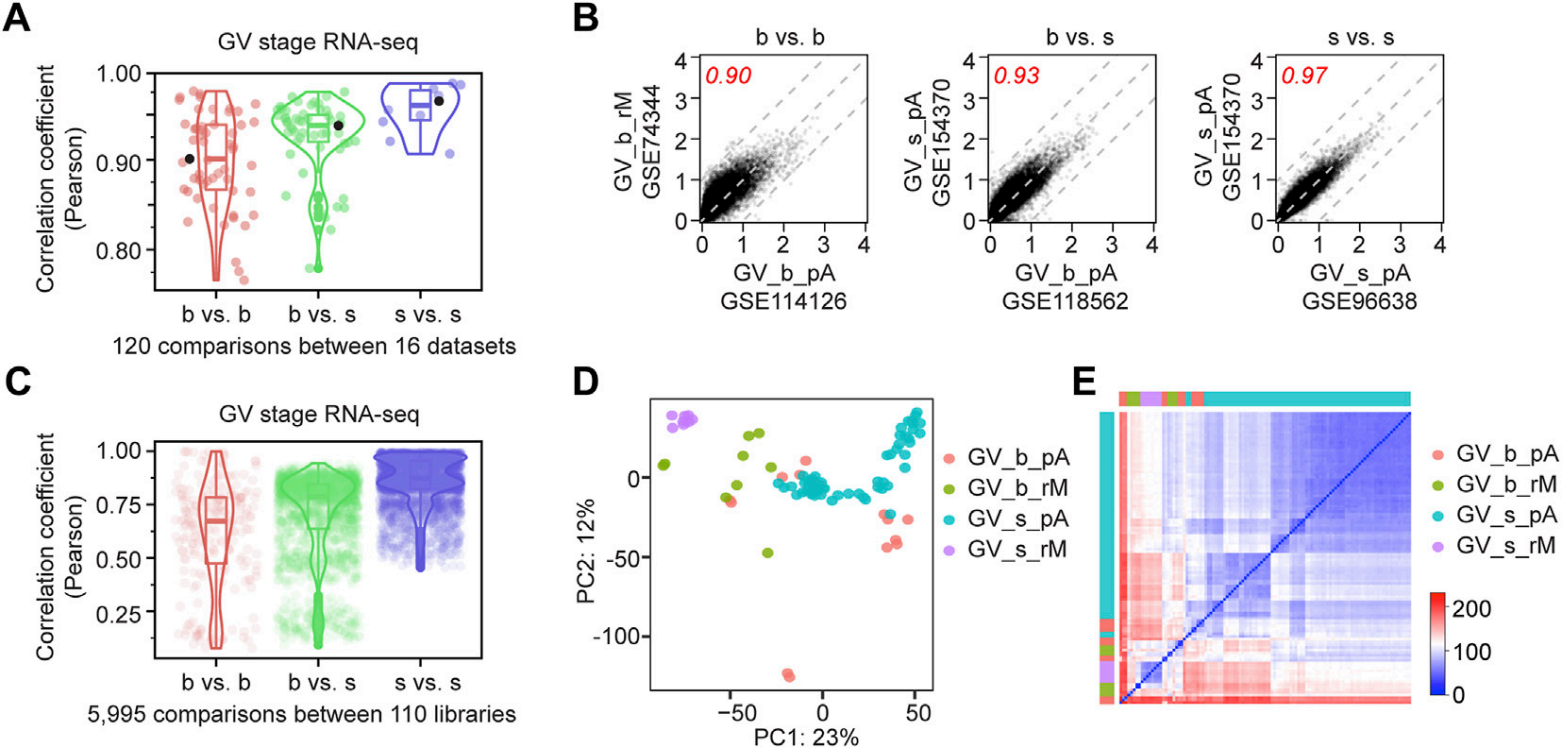
Single oocyte RNA-seq has high reproducibility. **(A)** Dot plots showing the Pearson correlation coefficients between 120 pair-wise comparisons of 16 RNA-seq datasets of mouse germinal vesicle (GV) oocytes. Dots are grouped by b vs. b (bulk vs. bulk), b vs. s (bulk vs. single) and s vs. s (single vs. single). Boxes and violins indicate the distribution of the dots. The three highlighted black dots are shown in **(B)**. **(B)** Examples from each comparison group in **(A)**, showing transcript abundance regression analysis between two datasets. Values represent the log_10_ mean of gene counts from all libraries in each dataset normalized by library sizes. Three gray dashed lines: y = x, y = x + 1 and y = x−1. Red numbers are the Pearson correlation coefficients. **(C)** Dot plots showing the Pearson correlation coefficients between 5,995 pair-wise comparisons of 110 individual libraries. Dots are grouped by b vs. b (bulk vs. bulk), b vs. s (bulk vs. single) and s vs. s (single vs. single). Boxes and violins indicate the distribution of the dots. **(D)** Principal component analysis of all 110 libraries from 16 datasets at GV stage, b: bulk, s: single, pA: poly (A) RNA-seq, rM: RiboMinus RNA-seq. Details of dataset information are in Supplementary Table S11. **(E)** Sample distance matrix of all 110 libraries from 16 datasets at GV stage.

The regression analyses of gene expression levels and ERCC molecules were performed by R (v3.5.1). For example, in Figure 1, when comparing between each library, the gene counts are normalized by the number of total counts; when comparing between each dataset (one dataset contains multiple libraries as biological replicates), the gene counts were firstly averaged across all libraries, then the obtained averaged gene counts were further normalized by the mean of total averaged gene counts.

### Use of Published Datasets for Ribosomal RNA (rRNA) Analysis

GSE141190 (Wu and Dean, 2020) contains ribosomal RNA sequencing datasets (rRNA-seq), which were performed using total RNA without rRNA depletion. Thus, majority of the reads come from rRNA due to the extremely high abundance of rRNA in a cell. GSE141190 dataset also contains poly (A)-based soRNA-seq libraries, which are used for analyzing the residual rRNA. For rRNA read analysis, the reads (from either rRNA-seq or poly (A) soRNA-seq) were directly mapped to and counted against the rRNA genome file (Grozdanov et al., 2003). Coverage values of each base in the rRNA genomic sequence was calculated using SAMtools coverage (v1.14) function. Heatmaps of rRNA coverage was plotted using plotnine (v0.8.0) in Jupyter-notebook. Line plot of rRNA coverage was plotted using seaborn (v0.11.2) in Jupyter-notebook. When calculating the coverage in each library, the coverage value of each position was firstly divided by the sum of coverage values of the entire rRNA transcript (from position 1–13,403), to get the normalized coverage values. Then the normalized coverage in the 5′-ETS region were summed to get the summarized coverage, which is used to color the dots in the PCA. Due to the existence of some spikes (Supplementary Figure S2B), the region used for 5′-ETS calculation was set to be 500–1,500 of 5′-ETS. Principal component analysis and k-means clustering were performed using the factoextra R package (v1.0.3).

## Results

### Single Oocyte RNA-Seq Exhibit Higher Reproducibility

To estimate the reproducibility of mouse oocyte RNA-seq, I obtained all published datasets from single or bulk mouse oocytes at GV (geminal vesicle) stage, which is the beginning of oocyte maturation (Xue et al., 2013; Kim et al., 2015; Veselovska et al., 2015; Yu et al., 2016a; Yu et al., 2016b; Dumdie et al., 2018; Guo et al., 2018; Sha et al., 2018; Yao et al., 2018; Seah et al., 2019; Taborska et al., 2019; Wu et al., 2019; Xu et al., 2019; Castillo-Fernandez et al., 2020; Du et al., 2020; Wu and Dean, 2020) (Supplementary Table S11). All raw sequencing files were processed in parallel. Different datasets have slightly different transcript distribution, and only the coding reads were taken for comparison (Supplementary Figures S1A–S1D; Supplementary Table S1). Interestingly, soRNA-seq datasets (GSE141190, GSE96638, GSE126688, and GSE44183) had higher correlation coefficients within the single group than when compared to the bulk group (Figures 1A–C). By unsupervised principal component analysis (PCA) that color-coded by datasets, single oocyte groups show higher similarity, though experiments/methods contribute to the observed differences (Figures 1D,E; Supplementary Figure S1E). Thus, soRNA-seq can generate highly reproducible and consistent results.

### Single Oocyte RNA-Seq Better Identify Populations of GV Oocytes

Fully grown mouse oocytes are highly uniform in their morphology and size. However, there are two different populations regarding nuclear configuration, developmental potential and transcriptional activity, namely non-surrounded nucleolus (NSN) and surrounded nucleolus (SN) oocytes (Ma et al., 2013; Schultz et al., 2018). Definitions of the two populations initially came from nuclear staining: NSN oocytes have puncta in the nucleus while SN oocytes have a circular signal surrounding nucleolus. Bulk RNA-seq have also revealed differences in NSN and SN oocytes (Ma et al., 2013). However, potential intermediate stages have been observed (Shishova et al., 2016), suggesting developmental progression at GV stage involves more complex changes. Separating the morphologically similar stages is technically difficult. I reasoned that soRNA-seq data would have an advantage to reveal the developmental progression compared to bulk RNA-seq. Through supervised clustering, soRNA-seq could demonstrate developmental stage progression.

**FIGURE 2:**
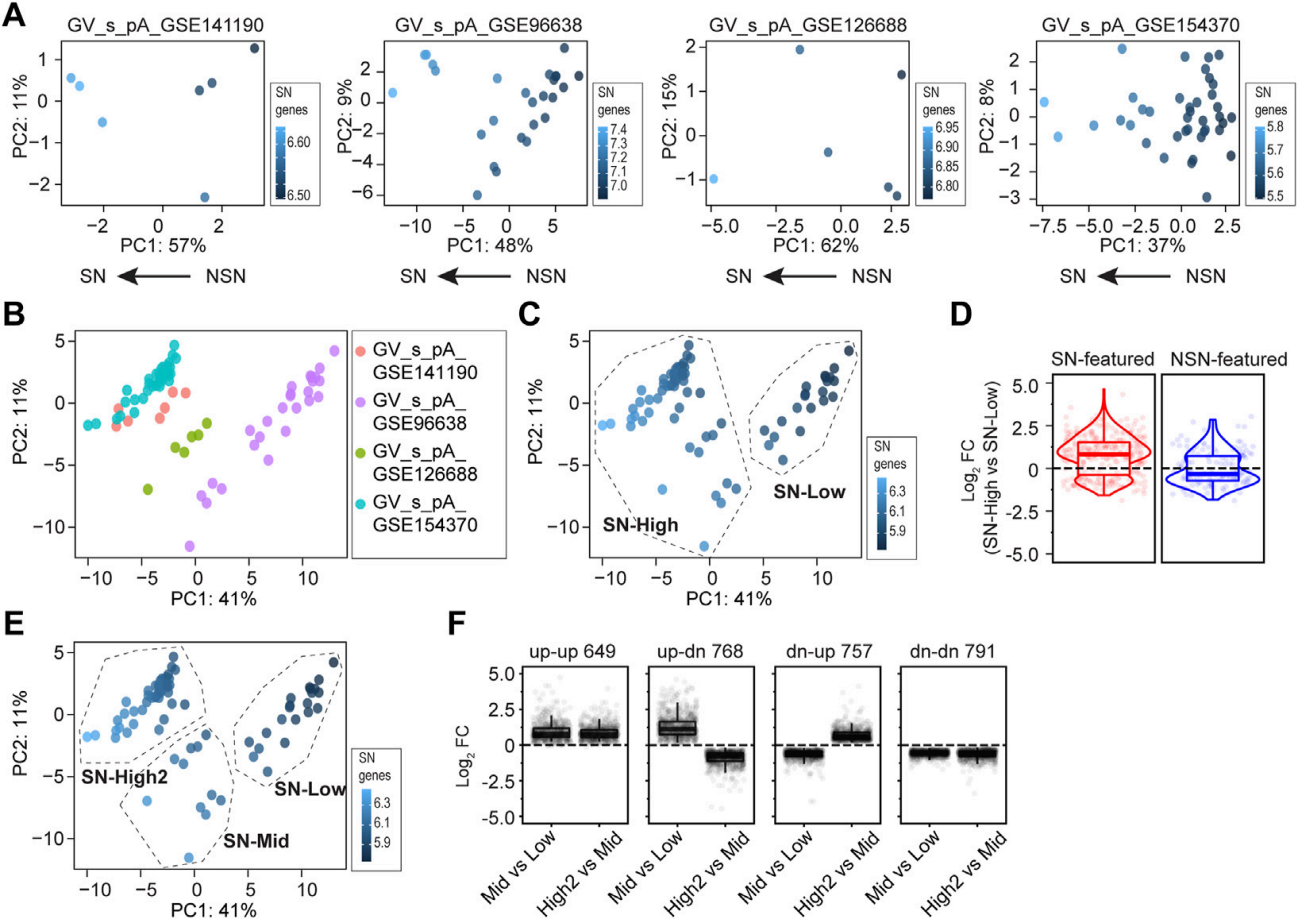
Single oocyte RNA-seq provides greater insight into GV oocyte development. **(A)** Principal component analyses of four single oocyte RNA-seq (soRNA-seq) datasets. In each plot, dots are color-coded by the mean count of pre-defined SN-featured genes (Ma et al., 2013). The arrows on the bottom suggest the developmental progression of all single oocytes within each dataset, identified by the level of the SN-featured genes. **(B)** Principal component analyses of combined datasets in **(A)** Dots are color-coded by the dataset identifiers. **(C)** K-means clustering based on **(B)**, with a pre-defined cluster number of 2. Dots are color-coded by the mean counts of SN-featured genes. SN-High and SN-low are assigned based on the level of the SN-featured genes. **(D)** Log_2_ fold change values of SN-High vs SN-Low in **(C)**. Each dot is a gene either SN-featured (shown in red) or NSN-featured (shown in blue). **(E)** K-means clustering based on B, with a pre-defined cluster number of 3. SN-High2, SN-Mid and SN-low are assigned based on the level of the SN-featured genes. **(F)** Dot plots showing log_2_ fold change values of SN-Mid vs. SN-Low, and SN-High2 vs. SN-Mid in **(E)**. Genes exhibit different changes from SN-Low to SN-Mid and from SN-Mid to SN-High2, including 649 genes that are up-regulated in both phases (up-up), 768 genes that are firstly up-regulated then down-regulated (up-dn), 757 genes that are first down-regulated then up-regulated (dn-up), and 791 genes that are down-regulated in both phases (dn-dn).

To document this, I analyzed all published soRNA-seq datasets (GSE141190, GSE96638, GSE126688, and GSE154370, respectively, Supplementary Table S11). Principal component analysis (PCA) of each dataset was conducted using defined SN-featured genes as the feature vector, which are the genes expressing more than two folds in SN oocytes compared to NSN oocytes (Ma et al., 2013). Then, the summarized counts of all SN-featured genes were profiled on the PCA plots. As a result, samples having higher level of SN-featured genes tend to have smaller PC1 values, which was further supported by the top five SN-featured genes individually (Figure 2A; Supplementary Figure S1F). Thus, each dataset can be assigned a developmental direction from NSN to SN along the negative PC1 axis (Figure 2A).

Next, I combined all four poly(A)-based soRNA-seq datasets (from GSE141190, GSE96638, GSE126688 and GSE154370, including 78 libraries in total) to allow more powerful analysis (Figure 2B). Based on the PCA using SN-featured genes, a k-means clustering was performed by pre-defining cluster number as 2. I named the two obtained clusters as “SN-High” and “SN-Low” according to the level of SN-featured genes in each cluster (Figure 2C). Presumably, SN-High cluster consists of oocytes more like the SN phase while SN-Low cluster consists of oocytes more like the NSN phase. As expected, most of the known SN-featured genes are up-regulated in SN-High group, while most of the NSN-featured genes are down-regulated in the SN-High group (Figure 2D) (Ma et al., 2013).

Then I sought to perform k-means clustering by defining cluster number as 3. Interestingly, the original SN-High cluster could be further divided into two subsets, which were named as SN-High2 and SN-Mid according to the level of SN-featured genes (Figure 2E). Presumably, SN-High2 consists of oocytes at very late GV stage, while SN-Mid consists of oocytes in the middle stage. Further differential analysis between the SN-High2, SN-Mid and SN-Low clusters revealed that many genes do not change consistently from NSN to SN. 768 genes firstly increase from early to middle stage and decrease during middle to late stage (up-dn). 757 genes firstly decrease from early to middle stage and increase during middle to late stage (dn-up). In addition, 649 genes consistently be accumulated from early to middle, and middle to late stage (up-up), while 791 genes consistently be cleared from early to middle, and middle to late stage (dn-dn) (Figure 2F; Supplementary Table S2). To summarize, using soRNA-seq datasets could obtain greater understanding of developmental progression of morphologically similar GV oocytes.

### Ribosomal RNA (rRNA) 5′-ETS Level Increases at SN Phase

**FIGURE 3:**
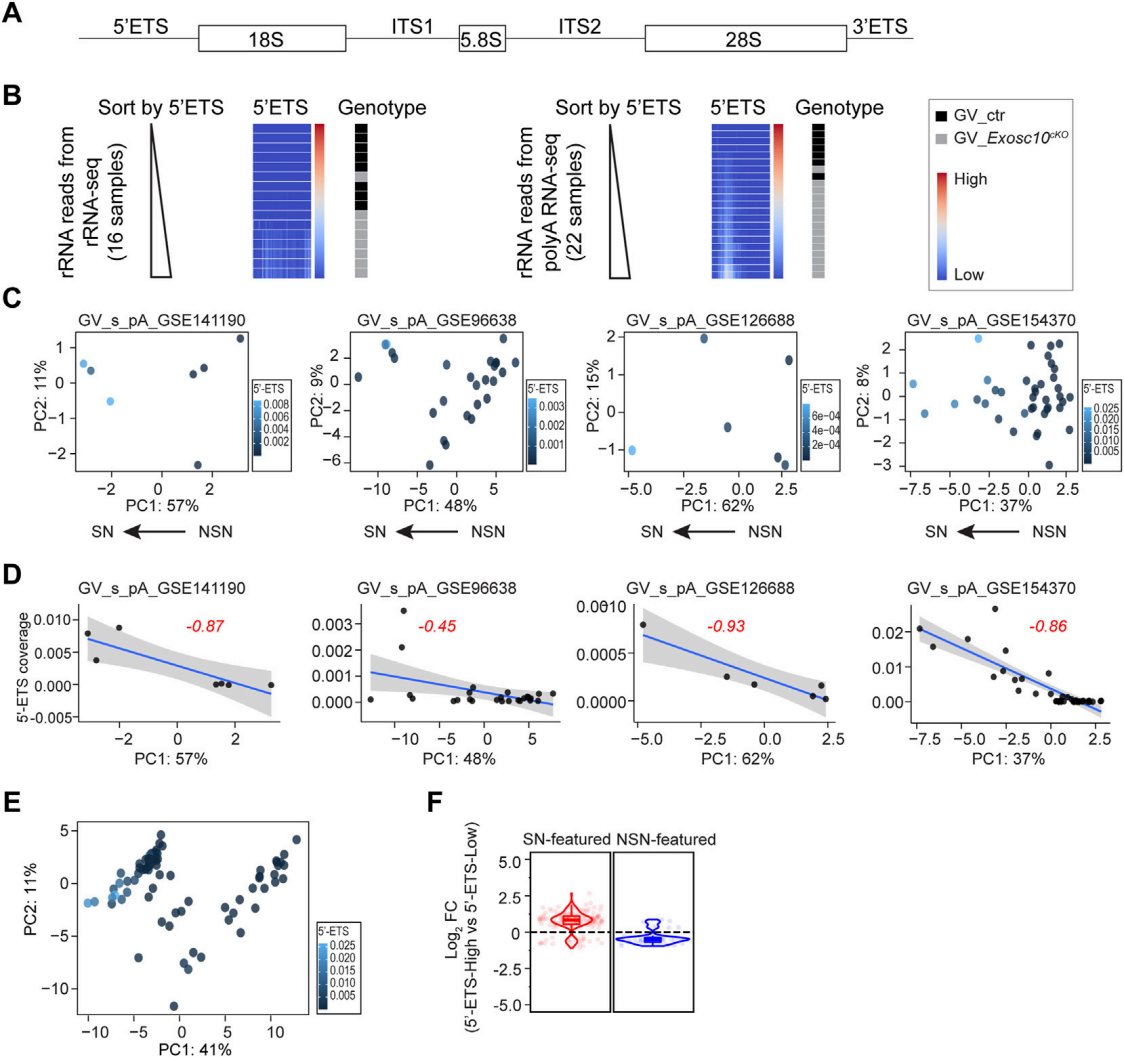
rRNA 5′-ETS elevation correlates with SN property. **(A)** Schematic of a mammalian rRNA primary transcript, including mature rRNA fragments (18, 5.8, and 28 S), external transcribed spacers (5′-ETS, 3′-ETS), and internal transcribed spacers (ITS1, ITS2). **(B)** Heatmaps of rRNA coverage document 5′-ETS up-regulation in EXOSC10-depleted oocytes. Left: rRNA reads obtained from rRNA-seq; right: residual rRNA reads obtained from poly (A) RNA-seq. Each heatmap is sorted in ascending order of the 5′-ETS coverage value. **(C)** PCA plots (the same as Figure 2A) color-coded by the 5′-ETS coverage values across all oocytes per dataset. **(D)** Regression analysis of PC1 and 5′-ETS coverage in each dataset. Blue line is the predicted linear regression line with 95% confidence interval. Red number is the Pearson correlation coefficient. **(E)** PCA plot (the same in Figure 2B) color-coded by the 5′-ETS coverage values across all oocytes. **(F)** Log_2_ fold change of 5′-ETS-High vs. 5′-ETS-Low in the combined four datasets in E. Pre-defined SN-featured and NSN-featured genes are shown in red and blue, respectively.

In eukaryotic cells, a primary rRNA transcript contains both mature rRNA subunit fragments, namely 18, 5.8 and 28S rRNA, and the Internal/External Transcribed Spacers, namely ITS1, ITS2 and 5′-ETS, 3′-ETS (Figure 3A) (Henras et al., 2015). Multiple cleavages and degradation take place to process the primary rRNA into mature 18, 5.8, and 28S rRNA fragments (Kent et al., 2009; Henras et al., 2015). Previous studies observed that rRNA processing happens differently in NSN and SN oocytes (Zhang et al., 2019). Depleting EXOSC10, an RNA exosome associated RNase in GV oocytes, can cause irregular rRNA pattern including elevated 5′-ETS and ITS1 (Wu and Dean, 2020). Interestingly, variation of 5′-ETS levels has also been observed in wildtype GV oocytes. I hypothesized that oocytes with variable 5′-ETS levels may correlate with different developmental status.

To substantiate this, I firstly evaluated whether the public soRNA-seq datasets are reasonable for rRNA 5′-ETS study by re-analyzing our own two datasets, including an rRNA-seq and a regular poly (A) soRNA-seq (from GSE141190, *Materials and Methods*), both of which contain EXOSC10 depleted oocytes and control oocytes. For both datasets, I extracted all reads that are mapped to rDNA genomic sequence and ranked the samples according to the 5′-ETS coverage (Figure 3B). As expected, the 5′-ETS from both rRNA-seq and poly (A) soRNA-seq show up-regulation in the EXOSC10 depleted oocytes. Thus, the 5′-ETS reads obtained from the residual rRNA reads in the poly (A) soRNA-seq can very well represent the real status of the rRNA in the original oocytes.

I then made use of the four poly (A) soRNA-seq datasets (GSE141190, GSE96638, GSE126688 and GSE154370, including 78 libraries in total). Due to the non-specific spike signals in the 5′-ETS, a region (500–1,500) was selected to calculate the 5′-ETS coverage, which in most datasets represent the residual 5′-ETS fragments (Supplementary Figure S2B, *Materials and Methods*). In each soRNA-seq dataset, I profiled the 5′-ETS coverage of all libraries in the PCA plot (Figure 3C). Regression analysis showed three out of four datasets exhibit a strong negative correlation between PC1 values and 5′-ETS coverage (coefficients of correlation as −0.87, −0.93, and −0.86, respectively, Figure 3D). Given that PC1 negatively correlates with SN status (Figure 2A), I concluded that SN oocytes likely have more 5′-ETS. Then the 78 oocytes were combined to define 5′-ETS-High and 5′-ETS-Low groups to perform differential analysis (the threshold is the mean value of the 5′-ETS coverage in all libraries, 0.00229). Strikingly, most of the SN-featured genes are up-regulated in 5′-ETS-High group, while most of the NSN-featured genes tend to be down-regulated (Figures 3E,F). A similar conclusion can also be obtained when I arbitrarily defined 5′-ETS-High and 5′-ETS-Low groups in each dataset (Supplementary Figures S2C,D). These finding indicates that elevated 5′-ETS may serve as a new biological feature of GV oocytes progression.

### Deduplication for Single Oocyte RNA-Seq Is Dispensable

**FIGURE 4:**
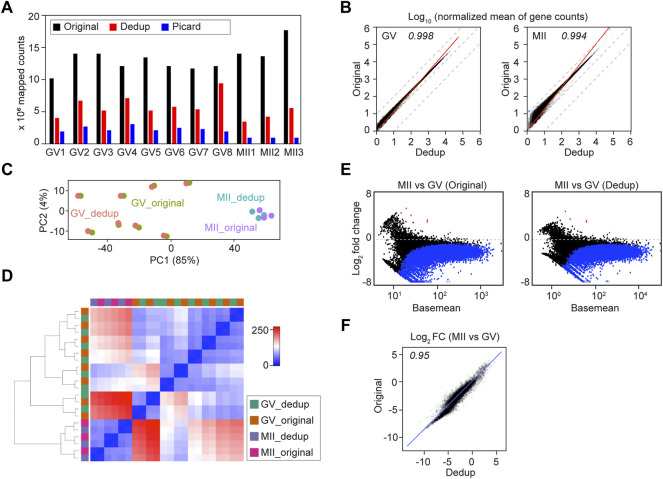
Deduplication of soRNA-seq reads does not significantly alter downstream analyses. **(A)** Bar graph of Original, UMI-deduplicated (Dedup) and Picard-deduplicated counts. GV1-GV8, MII1-MII2 are oocyte IDs from GSE141190 RiboMinus datasets. **(B)** Plots of transcript abundance regression analysis of Original vs. Dedup gene counts. The x-axis and y-axis are log_10_ normalized mean counts from Original and Dedup. **(C,D)** Principal component analysis **(C)** and sample distance matrix **(D)** of combined Original and Dedup. **(E)** Differential analysis of MII vs. GV in Original and Dedup. **(F)** Comparison of the log_2_ fold change (MII vs. GV) in Original and Dedup. The x-axis and y-axis are log_2_ fold change (MII vs. GV) values in Original and Dedup.

Single cell RNA-seq is susceptible to many biases, including gene capture, reverse transcription efficiency and amplification cycles (Hwang et al., 2018). The incorporation of UMI (unique molecular identifiers) significantly improves single cell sequencing reproducibility by quantifying reads with more precision (Islam et al., 2014). To test whether UMI also benefits soRNA-seq, I re-analyzed the GSE141190 RiboMinus RNA-seq using UMI deduplication which determines duplicates by both UMI and alignments. The N8 UMI, capable of distinguishing up to 65,536 molecules, is sufficient to distinguish the ∼20,000 different RNAs expressed in mouse oocytes (Piko and Clegg, 1982; Wu and Dean, 2020). On average, the number of reads of the Dedup (UMI-based) samples was 43% ± 15% of their Original samples (Figure 4A; Supplementary Figure S3A; Supplementary Table S3). After filtering out low-count genes, the linear regression of gene counts in Original and Dedup groups, normalized by library size, exhibited a high correlation at the same stage (Figure 4B).

Then I performed downstream differential analysis using both Original and Dedup counts. The Original and Dedup counts from the same oocyte were similar, documented both by unsupervised PCA and hierarchical clustering of sample distances (Figures 4C,D). The MII vs GV differential analysis in both Original and Dedup groups also were very similar (Figures 4E,F). On the other hand, no significant change in gene expression was identified (*P*-adjusted < 0.01) when comparing Original and Dedup at the same stage (Supplementary Figure S3B).

To further evaluate how important deduplication is to single oocyte RNA-seq, I took advantage of Picard deduplication, which defines duplicates based only on mapping coordinates. As expected, Picard trimmed off even more reads (Figure 4A). Nevertheless, the correlation between Original and Picard remained high though obvious deviation was present due to read trimming (Supplementary Figure S3D). As expected, differential analyses of Original vs Picard at the GV or MII stages documented that a certain group of genes, including those encoding ribosomal proteins (*Rpl19*, *Rpl32*, *Rps20*) were sensitive to deduplication (Supplementary Figure S3C; Supplementary Table S4). In addition, I took advantage of the ERCC (External RNA Controls Consortium) spike-in molecules, which were added to oocytes at the beginning of library construction, to visualize the linearized amplification of the libraries. All Original, Dedup and Picard groups had comparable numbers of ERCC molecules detected, and all generated a high correlation of ERCC molecule counts with their concentrations (Supplementary Figures S3E–G). This suggests that under a reasonable library amplification condition, the RNA molecules being detected, and their relative levels remain the same with or without deduplication. Thus, deduplication provides only a limited advantage to the robustness of soRNA-seq, possibly due to the high RNA abundancy that makes the oocyte more like a tissue (bulk) rather than a standard single cell.

### Identification of Constant Genes for Cross-Stage Normalization

It has been long known that around 80% transcriptome undergoes dramatic decrease during mammalian oocyte maturation (Piko and Clegg, 1982; Yu et al., 2016a; Wu and Dean, 2020). Presumably, identification of the decreased and stable RNA in this process could provide insight into the molecular regulation of oocyte development and oocyte quality control. Several RNA-seq readouts have been used to represent cross-stage differences such as FPKM (Fragments Per Kilobase Million) and RPM (Reads Per Kilobase Million) which are normalized by gene length and library size (Yu et al., 2016a; Zhang et al., 2018). However, this library-size based quantification indicated as many genes with increased abundance as with decreased abundance, which is difficult to explain biologically in the absence of transcription during oocyte maturation. Gfp/Rfp spike-in molecules have also been used to quantify changes in the transcriptome size, but not for differential analysis of individual genes (Yu et al., 2016a).

Here I propose that the ERCC spike-in mix can serve as control genes for soRNA-seq. In all 89 soRNA-seq libraries, 31 libraries having ERCC reads more than 500 were extracted of both GV and MII stages, including 15 from GSE141190 poly (A), five from GSE96638 poly (A) and 11 from GSE141190 RiboMinus (Supplementary Table S11). When computing sample distances, using ERCC normalization exhibited more variation within GV stages, but the GV and MII stages can be very well separated compared to the median ratio normalization (Supplementary Figure S4A). In addition, ERCC normalization can better reflect the RNA degradation happening during meiotic maturation (Supplementary Figure S4B).

**FIGURE 5:**
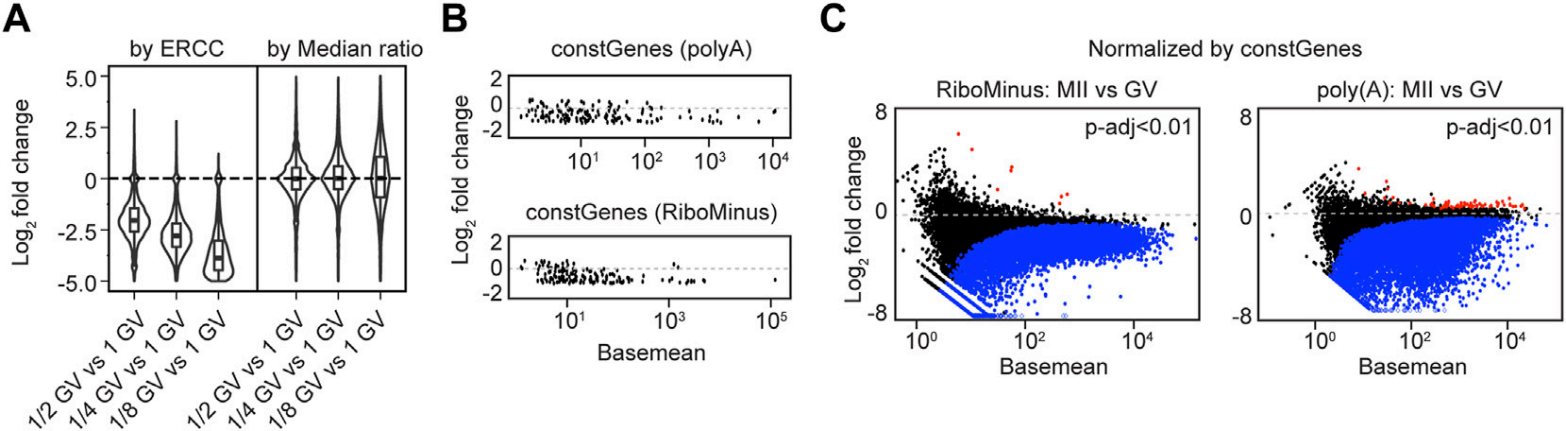
Applying constant genes (constGenes) for cross-stage comparison. **(A)** Summaries of log_2_ fold change values in fractional (1/2, 1/4, 1/8) GV oocyte vs. 1 GV oocyte by ERCC normalization and median ratio normalization. **(B)** MA plots of selected 147 constant genes (constGenes) from poly (A) and RiboMinus RNA-seq results. **(C)** MA plots showing the differentially expressed genes (MII vs. GV) from RiboMinus RNA-seq and poly (A) RNA-seq, normalized by constGenes. Blue/red dots: genes having decreased/increased abundance by *P*-adjusted < 0.01.

To better illustrate ERCC normalization, I performed poly (A) RNA-seq of GV oocytes using different fractional amounts (1/2, 1/4, 1/8) of whole oocytes. The differential analysis also confirmed the overall smaller library sizes of the fractional GV oocytes compared to the intact GV oocyte (Figure 5A; Supplementary Figures S4C,D; Supplementary Tables S5, S6). Thus, I conclude that exogenous spike-in accounts for changes in library size and facilitates investigation of transcriptome degradation during oocyte maturation.

To allow cross-stage comparison of sequencing samples when ERCC is unavailable, I have extracted a set of 147 constant genes (constGenes) during oocyte maturation. These 147 genes exhibit little change (less than 50%) in transcript abundance from GV to MII whether obtained by poly(A) RNA-seq or RiboMinus RNA-seq (Figure 5B; Supplementary Table S7). The constGenes span a large range of gene lengths (∼0.5–27 kb), have GC content ∼30%–60%, contain protein coding genes and lncRNAs (Supplementary Table S8). As expected, the differential analysis normalized by constGenes was very similar to those normalized by ERCC (Figure 5C, Supplementary Figures S4E; Supplementary Tables S9, S10).

In summary, based on higher reproducibility and known genetic/developmental heterogeneity, single cell RNA-seq appears to be a better method for transcriptome analyses of mouse oocytes. When an overall change of transcriptome size is anticipated, external spike-in or the constGenes are recommended for normalization to provide better insight into biological changes of the transcriptome.

## Discussion

This study has compared currently available mouse GV oocyte RNA-seq datasets and concluded that soRNA-seq has high reproducibility and reveals better details of developmental progression. In addition, this study has also established external spike-in or constant genes normalization method for cross-stage comparison, and suggests that deduplication of the single oocyte RNA-seq datasets does not significantly alter the downstream analyses.

A fully grown oocyte has much larger size and more RNA content compared to a somatic cell. This feature makes a single oocyte a unique type of single cell, which is more like a “bulk” status. For example, a regular somatic cell could capture around a few thousand genes using 10x genomic sequencing (Wang et al., 2019), or around 10,000 genes using SMART-seq (Tang et al., 2009), while a single GV oocyte could detect up to 20,000 genes to the same scale as oocyte bulk RNA-seq 50–100 oocytes pooled as one sample). From our pair-wise comparison (Figure 1A), it is surprising that though pooled oocytes may have better reproducibility between replicates in one dataset (Supplementary Figure S1E), the single oocyte RNA-seq generally have better correlation across different datasets. This may be due to the relatively uniform sequencing methods for single oocyte RNA-seq. Secondly, duplication level in libraries is directly determined by the number of amplification cycles, sequencing depth, and initial material. Within a reasonable amplification range (i.e.,10–18 cycles in GV oocytes), duplication level is not detrimental (data not shown), making deduplication dispensable. When even more amplification cycles are used, deduplication could be critical.

Unlike different cell types detected in a tissue-derived single cell RNA-seq, single oocytes have almost identical morphology and size. Thus, the heterogeneity comes mostly from developmental variation or potential quality difference, which has been largely neglected in bulk RNA-seq. By integrated analysis of all soRNA-seq datasets, genes that undergo increase or decrease during early or late phases of GV oocytes have been discovered, which provide more information than the NSN and SN-featured genes identified by bulk RNA-seq. Presumably, under conditions that could cause more variation, such as chemical treatment or genetic perturbation, employing soRNA-seq can better illustrate the spectrum of changes in oocytes. In addition, I observed rRNA 5′-ETS level exhibits high correlation with SN property, which may be caused by both rRNA transcription during meiotic prophase I and the different processing mechanisms of individual rRNA fragments. Given the extremely low level of rRNA 5′-ETS in a cell, it is hard to setup a threshold to define SN phase. However, when multiple single oocytes are compared, the earlier and later stages could be very well aligned based on their rRNA 5′-ETS levels.

In summary, this study demonstrates the advantage of soRNA-seq. I have also proposed rRNA 5′-ETS as a new marker for SN oocytes and established constant genes-mediated cross-stage normalization.

## Data Availability

The datasets presented in this study can be found in the Gene Expression Omnibus website with accession code GSE145283. The accession numbers of all other published repositories used in current study can be found in Supplementary Tables.
