# Supplementary figures and images for: Mouse Oocytes, A Complex Single Cell Transcriptome
Source: Front Cell Dev Biol. 2022 Mar 7;10:827937. doi: 10.3389/fcell.2022.827937 (PMC8935041; doi:10.3389/fcell.2022.827937)

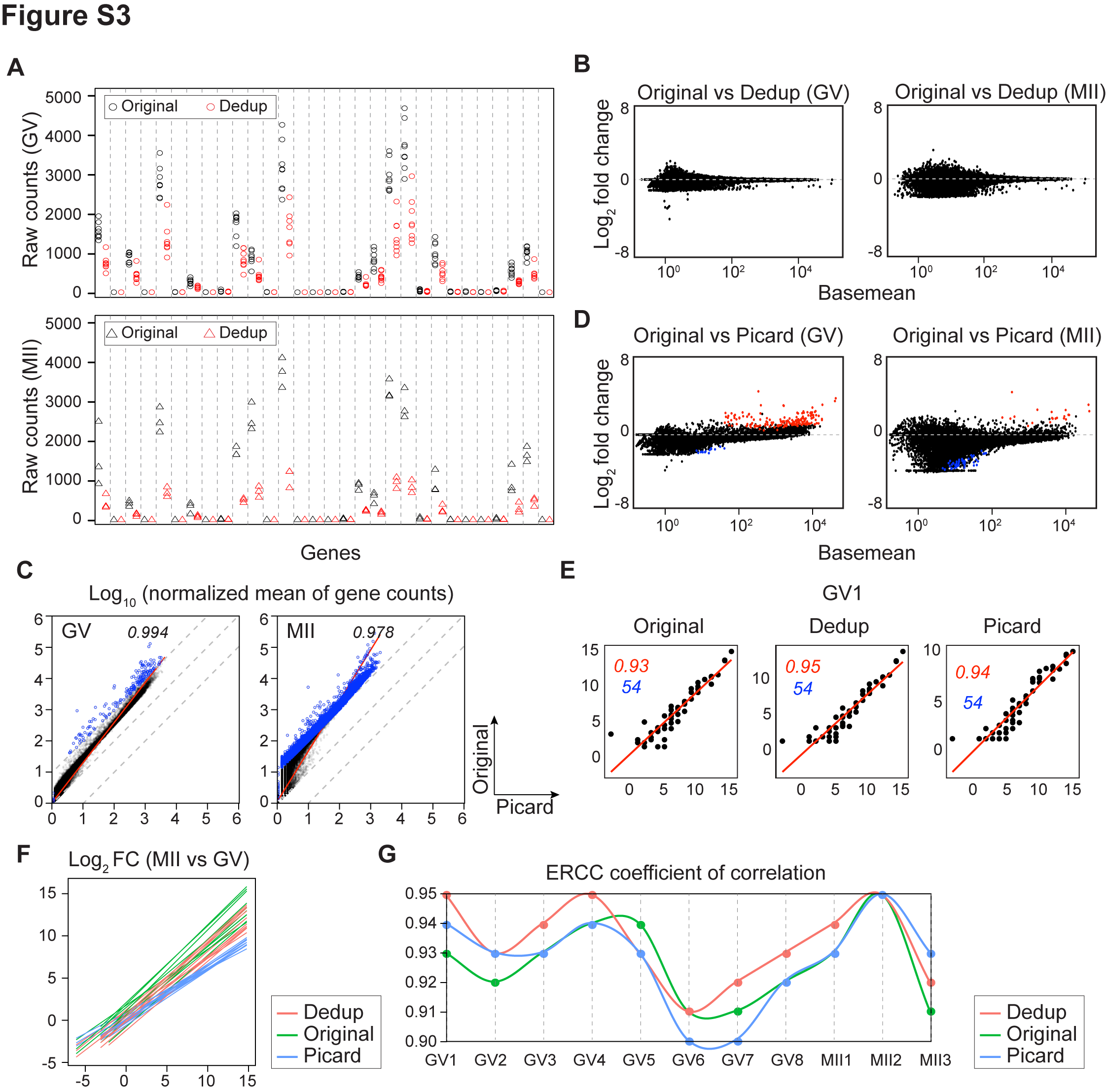

Supplement: Supplementary file 4 [file Image3.TIF]

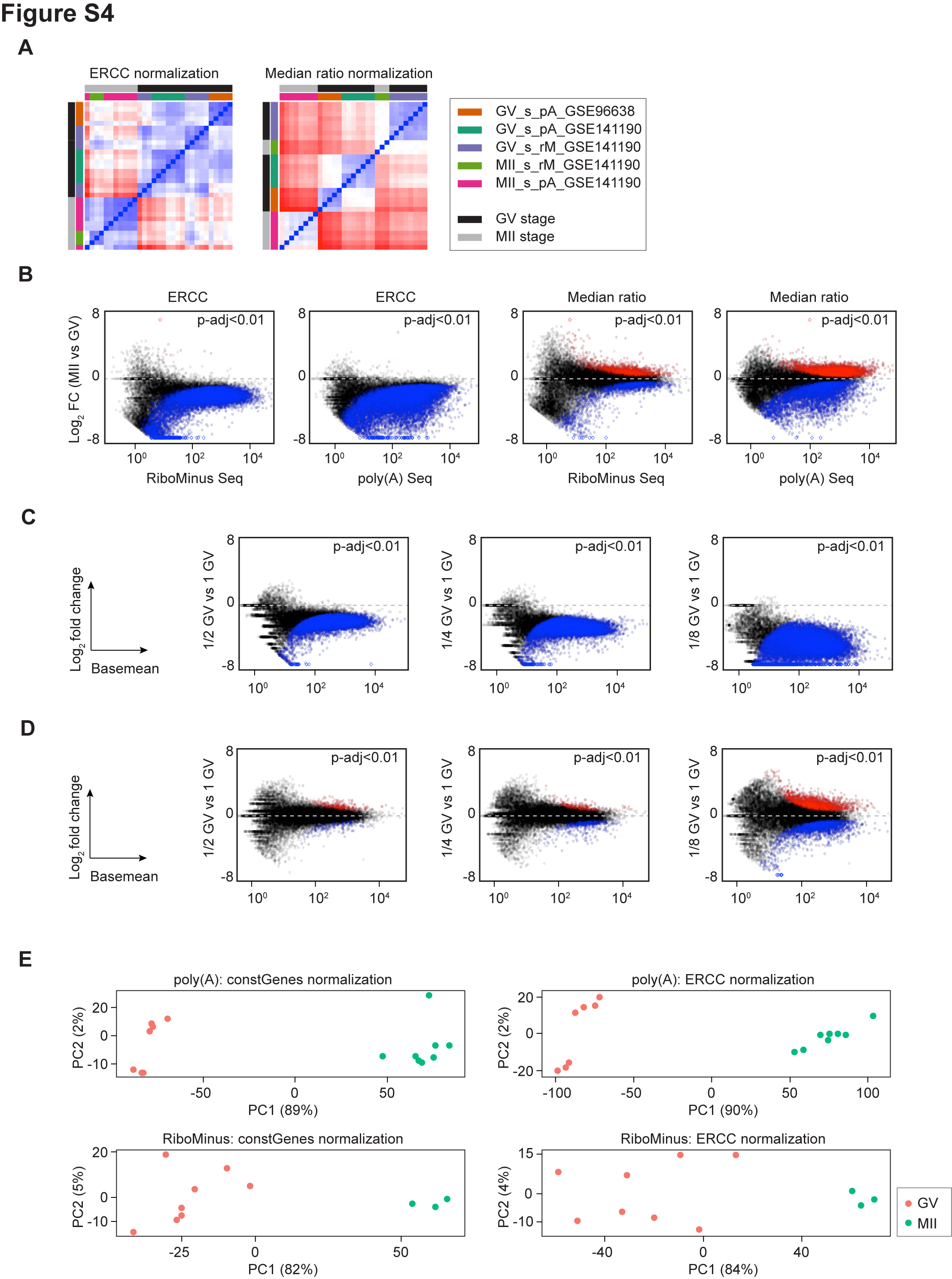

Supplement: Supplementary file 5 [file Image4.TIF]

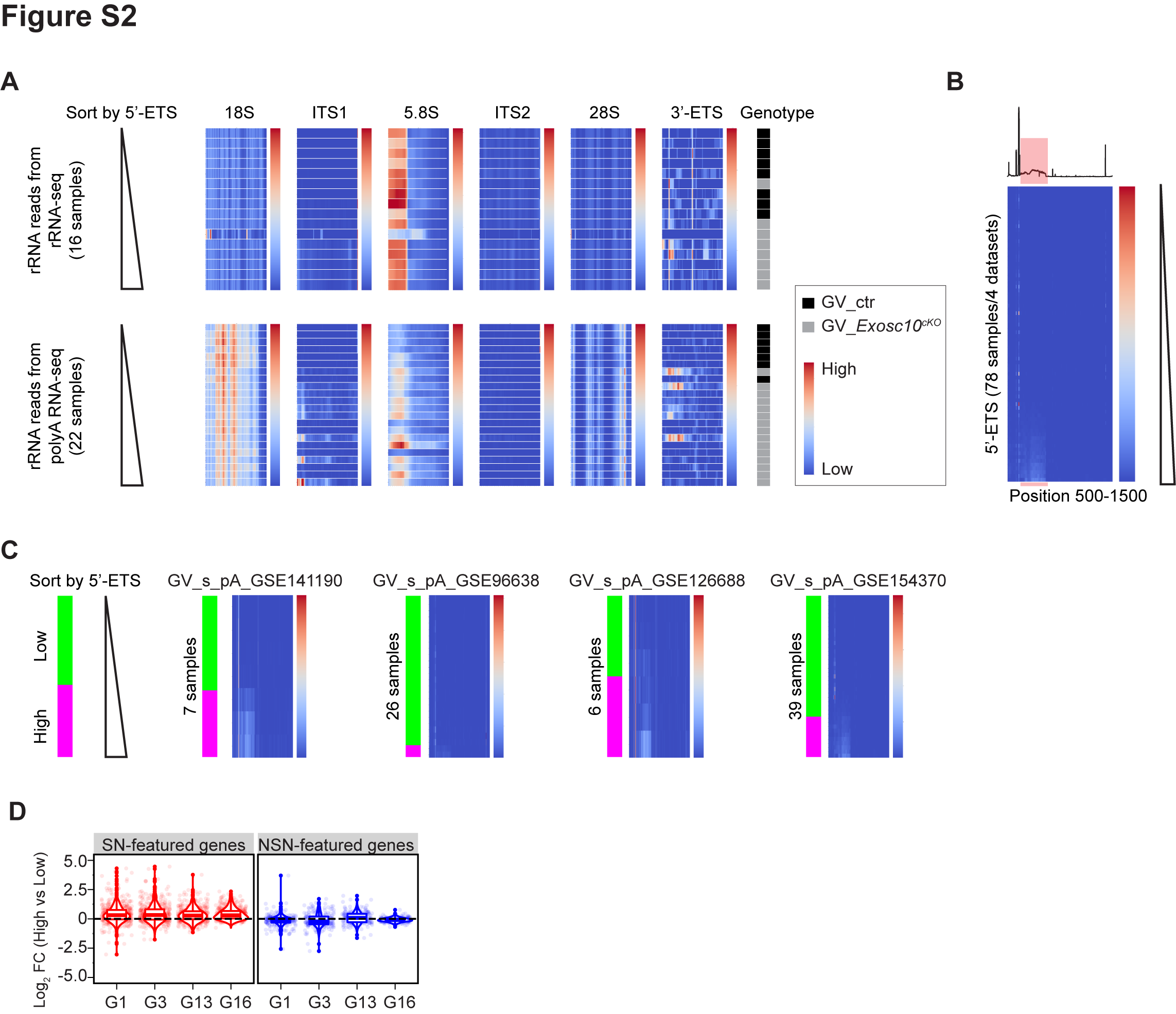

Supplement: Supplementary file 6 [file Image2.TIF]

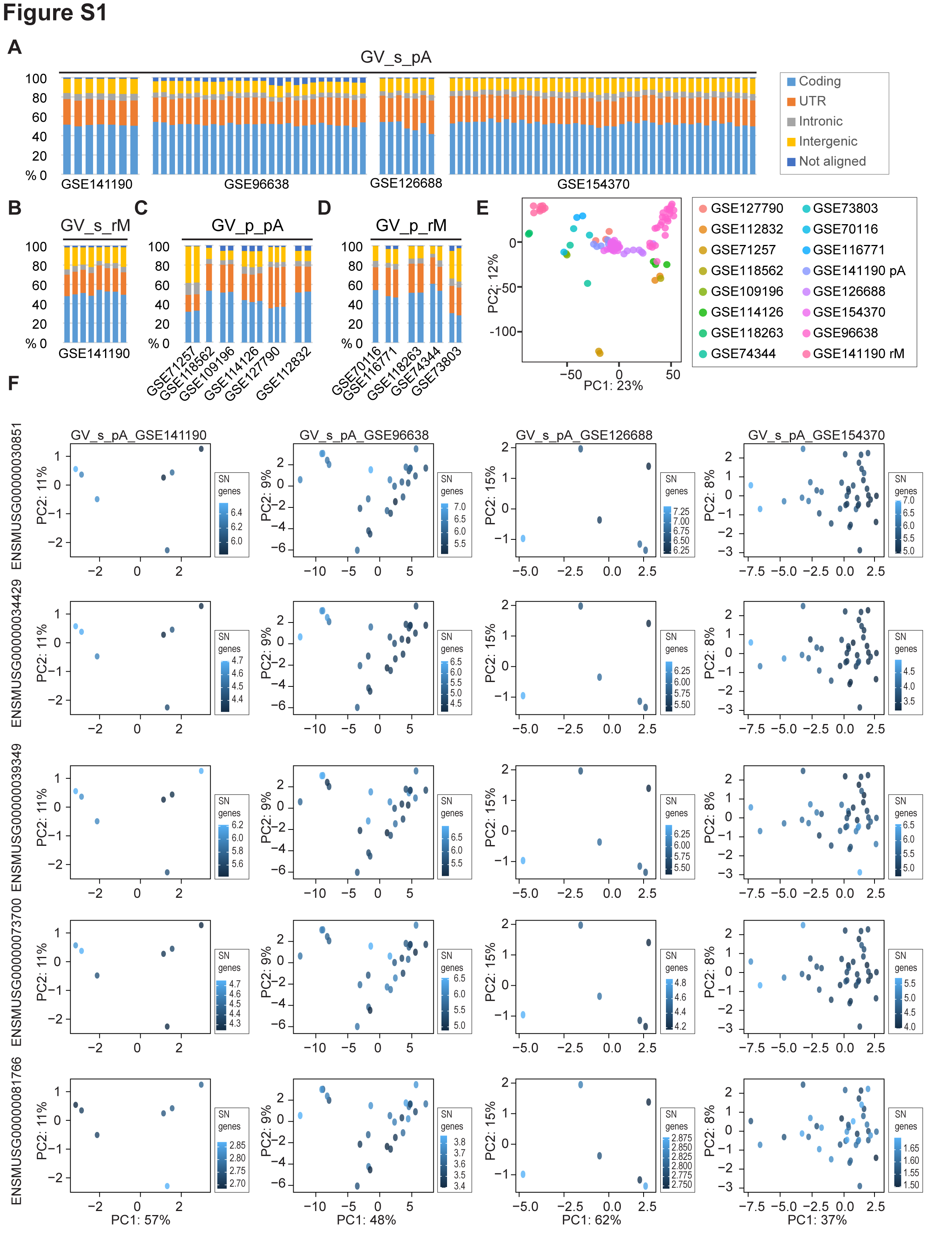

Supplement: Supplementary file 7 [file Image1.TIF]
